# Overview of Common Sleep Disorders and Intersection with Dermatologic Conditions

**DOI:** 10.3390/ijms17050654

**Published:** 2016-04-30

**Authors:** Harneet K. Walia, Reena Mehra

**Affiliations:** Center for Sleep Disorders Cleveland Clinic 11203, Stokes Blvd Cleveland, Cleveland, OH 44195, USA; mehrar@ccf.org

**Keywords:** sleep disorders, insufficient sleep, sleep and skin disorders

## Abstract

Sleep disorders are very common, often under-recognized and therefore undertreated, are associated with a myriad of medical conditions and could lead to significant impairment of quality of life. This review provides an up-to-date synopsis of common sleep disorders encompassing insufficient sleep syndrome, insomnia, circadian rhythm disorders and obstructive sleep apnea with a brief overview of epidemiology, screening, diagnostic testing and treatment. We also emphasize the emerging area of the intersection of sleep disorders and dermatologic conditions and present compelling data regarding underlying mechanisms including sleep dysfunction in relation to disorders of skin inflammation, aging and skin cancer.

## 1. Introduction

An estimated 50–70 million American adults suffer from one or more sleep disorders [1]. There are approximately eighty different types of sleep disorders. Notwithstanding the fact that sleep is vital to human health [2], the sleep disturbances that many individuals suffer from often do not come to clinical attention. As a result, many Americans chronically suffer from sleep disorders impacting their daytime functionality and health. Factors that lead to under-detection of sleep disorders include lack of recognition of the relevance of sleep problems by patients and lack of awareness of the clinicians concerning evaluation and management sleep problems. As almost all medical specialties intersect with sleep disorders directly or indirectly, sleep medicine is inherently interdisciplinary. Moreover, a number of medical and surgical specialties including neurology, pulmonary medicine, psychology, psychiatry, pediatrics, otolaryngology and dentistry are integral to the multidisciplinary, comprehensive management of sleep disorders. Disorders of sleep interrelate with a variety of disease states, with emerging data now underscoring the importance of sleep disorders and disturbances of sleep as it relates to dermatologic conditions. The impact of sleep disorders can have far-reaching health implications including increased risk of drowsy-driving-related motor vehicle accidents, increased risk of a broad range of chronic disease states such as hypertension, diabetes mellitus, obesity, cardiovascular disease, depression and even cancer, and several that also serve to increase mortality risk [1]. In recent years, the impact of untreated sleep disorders has become increasingly recognized and clinicians will certainly require improved knowledge in the realm of sleep disorders to adopt measures to improve the recognition, diagnosis and treatment of some of the most common sleep problems, particularly as untreated sleep disorders can adversely impact health. Here we will review common sleep disorders, *i.e.*, insufficient sleep syndrome, insomnia, circardian rhythm disorders and obstructive sleep apnea, as well as their intersection with dermatologic conditions to provide a state-of-the-art update for clinicians in an effort to increase awareness about the importance of sleep disorder recognition and treatment.

## 2. Sleep Deprivation

Insufficient Sleep Syndrome, classified as one of the sleep disorders in the International Classification of Sleep Disorders -3 (ICSD-3) [3], may alternatively be referred to as chronic sleep deprivation, sleep restriction and behaviorally induced insufficient sleep syndrome. The adage of “can’t sleep versus won’t sleep” helps to distinguish insufficient sleep syndrome from insomnia, whereby sleep deprivation is characterized by the decision to not obtain sufficient sleep, *i.e.*, due to lack of prioritization, and alternatively insomnia is characterized by the inability to obtain sleep or having a sense of unrefreshing sleep when other etiologies have been excluded. Typically, with extension of the total sleep time there is resolution of symptoms of sleepiness associated with sleep deprivation. Center of Disease Control and Prevention (CDC) data from 1985–2012 showed a major decline in sleep duration among individuals to less than 6 h [4], an impressive and concerning observation resulting in the assignment of sleep deprivation as a public health epidemic. Furthermore, a recent CDC MMWR statement based on surveys collected from 444,306 people in 2014 highlighted the high prevalence of sleep deprivation, *i.e.*, one in three Americans do not obtain sufficient sleep [5]. The dramatic upswing in the prevalence of sleep deprivation likely has a multifactorial basis including socioeconomic demands and increased light exposure to meet the needs of modern society.

Sleep deprivation is associated with cognitive impairment [6] as well as with daytime sleepiness and a negative impact on cognitive performance [7]. Furthermore, it affects productivity negatively increasing the risk of occupational errors and auto accidents [8]. In fact, even one night of deprived sleep can impair performance equal to blood alcohol content of 0.10 percent; *i.e.*, above the legal limit to drive.

Results of a recent population based study indicate that young adults who slept less than 7 h were more likely to report poor general health and lower quality of life than those who slept more than 7 h of sleep [9]. An array of adverse cardiovascular outcomes is associated with short sleep duration.

A prospective study involving young adults showed that short sleep duration was associated with incident hypertension even after adjustment for confounders including obesity [10]. Another study reinforced that decreased total sleep time is associated with increased risk of incident hypertension [11]. In a large study involving approximately 30,000 individuals, those who slept less than 5 h had twice higher odds of having heart disease including myocardial infarction and stroke compared to those who slept 7 h per night [12]. A large prospective study with a follow up over a decade showed 23% greater risk of coronary artery disease than those who slept less than 7 h a night [13].

In terms of overweight/obesity, sleep restriction has been shown to increase hunger and appetite and craving for fat and sweet snacks thereby resulting in increased body weight [14]. This is potentially attributed to reductions in leptin levels and increases in ghrelin levels. Recent data suggests alterations of the endocannabinoid system may contribute to the increased drive of food intake in a sleep-deprived state and has emerged as a novel contributory factor for obesity [15]. Another recent study showed that two nights of recovery sleep were able to revert the effects of short term sleep restriction on diabetes parameters [16]. Furthermore, sleep loss is considered to be a risk factor for insulin resistance and type 2 diabetes [17]. Some of the deleterious effects of sleep deprivation are related to impairment in the hypothalamic-pituitary axis activation resulting in elevation of cortisol levels [9]. Effects of sleep deprivation on the immune state are also evidenced by data suggesting that there is heightened susceptibility to common cold to those who are sleep deprived [18].

### Addressing Sleep Deprivation

A recent joint consensus statement from the American Academy of Sleep Medicine and the Sleep Research Society has provided guidelines on the recommended amount of sleep requirements [19]. The consensus states that adults aged 18–60 years should sleep 7 or more hours per night on a regular basis to promote optimal health and wellbeing [20]. However, these guidelines do not address other dimensions such as timing, regularity and quality of sleep.

## 3. Insomnia

Insomnia, one of the most common sleep disorders, is defined as difficulty falling sleep (sleep onset insomnia), maintaining sleep (sleep maintenance insomnia) or poor quality sleep despite adequate opportunity/circumstances for sleep and results in daytime impairment. The prevalence of insomnia varies with the study design and type of population studied. A review of 50 studies showed the prevalence of insomnia to be 10% [21]. Transient symptoms of insomnia can occur in 30%–35% of the population [22]. The prevalence of insomnia increases with increasing age and is found to be more prevalent in women [23] and family history represents another insomnia risk factor suggesting heritability [24]. Insomnia commonly occurs in the context of psychiatric disorders, characterized by a prevalence of ~40%–50%, occurring in particular in mood and anxiety disorders.

The recent ICSD-3 [3] classification has identified insomnia subtypes based on the chronicity of symptoms, *i.e.*, chronic insomnia disorder, short term insomnia disorder and other insomnia disorder. Chronic insomnia is defined as sleep disturbance for at least three months and symptoms that occur at least three times per week [25]. The definition considers the fact that the sleep complaint is accompanied by consequences impairing family, social or academic life. The patients suffering from chronic insomnia often focus excessively on their inability to sleep and daytime consequence associated with it. Short term insomnia has been called acute insomnia, transient insomnia or adjustment insomnia. The symptoms are generally present for less than three months and transiently associated with a stressor. It generally resolves with the resolution of a stressor.

Insomnia can cause impairment in quality of life ranging from excessive sleepiness, anxiety and depression. Also patients who are not able to obtain treatment from their clinicians tend to self-medicate and are at increased risk of substance abuse. Furthermore, about quarter of patients with insomnia have tried alcohol as a treatment for insomnia [26].

Insomnia and mood disorders share a bidirectional relationship. In a population-based study involving approximately 8000 individuals, 40% of those with insomnia had a psychiatric disorder [27]. Difficulties with sleep onset in adolescent years are associated with increased depression risk in later life [28]. Insomnia is considered a predisposing factor for psychiatric disorders including depression and anxiety [29]. It is estimated that 15%–20% of people with insomnia will have major depression [30].

Furthermore, insomnia has been shown to be associated with objective, physiologic abnormalities consistent with the hyperarousal state, including sympathetic nervous system activation, altered heart rate variability and increased EEG beta activation, and some data support relationships of insomnia as a risk for hypertension [31] and myocardial infarction. Persistent insomnia has been shown to have increased all-cause and cardiopulmonary morality in large observational studies, which persisted even after adjusting for systemic marker C-reactive protein [32]. Up-regulation of systemic inflammation is a purported mechanistic pathway underlying the association of insomnia and cardiovascular disease.

### 3.1. Treatment of Insomnia

An extensive sleep history including sleep-wake timing during the weekdays and weekends including daytime napping should be obtained. Other habits involving lifestyle history should be ascertained—for example, frequency, amount and timing of caffeine intake.

Patients suffering from psychiatric conditions, medical conditions or sleep disorders as a root cause of insomnia should receive optimization of treatment for these respective conditions. Although pharmacologic agents may be used for the treatment if insomnia, e.g., sedative hypnotics, there are sufficient evidence-based data to support the use of non-pharmacologic approaches which may be as efficacious and durable [33]. The behavioral therapy approach for insomnia treatment includes sleep hygiene education, stimulus control, sleep restriction, cognitive therapy and relaxation techniques. A recent systematic review and meta-analysis included 1162 participants incorporating cognitive therapy, stimulus control, sleep restriction and sleep hygiene; significant improvement in sleep onset latency (time to sleep onset) was observed, wakefulness after sleep onset time decreased and total sleep time and sleep efficiency improved. These changes were sustained at later time points [34].

General sleep hygiene education that is part of the standard in the care of the patient with a sleep disorder includes tenets such as maintaining a regular sleep wake schedule even during the weekends and days off of work, avoidance of caffeine within 4–6 h prior to sleep time and avoidance of daytime naps if possible, the latter to allow for sufficient sleep pressure to achieve preferred sleep during the night-time hours.

### 3.2. Circadian Rhythm Disorders

Circadian rhythm disorders occur when there is misalignment between the internal rhythm and the required timing related to the patient’s social obligations.

Delayed Sleep Wake Phase Disorder (DSPD) is one such example which is most common in adolescents and young adults [35] and characterized by a delay in the major episode sleep period in relation to required or desired sleep and wake up time with an estimated prevalence of 0.2%–10%. This is evident by difficulty falling asleep and difficulty awakening at a desired clock time [3]. When allowed to follow one’s preferred schedule, sleep quality and quantity are reported as normal. Long-lasting DSPD can affect quality of life adversely [36]. In addition, DSPD is also linked with loss of jobs and school failure [37]. The key etiology of DSPD is significant delay in timing of circadian rhythm [38]—it has been reported that there is a delay of 2–6 h in DSPD in circadian phase timings compared to normal sleepers [39]. DSPD patients have a delay in the timing of the core body temperature minimum, the latter closely tied with circadian rhythm timing [39]. Correspondingly, the peak level of melatonin, a hormone made by the pineal gland which controls the sleep-wake cycle, occurs approximately 4 h later in DSPD patients compared to controls [40]. Other etiological process considered include a longer circadian period which means that those with DSPD may take an increasingly longer time to complete the full circadian cycle characterized by a longer circadian period (tau) [41]. It is also postulated that those with DSPD accumulate sleep drive gradually compared to controls with slow dissipation during sleep [42]. Moreover, personality and psychosocial factors contribute towards DSPD as well [43].

Advanced Sleep Phase Disorder (ASPD) is when the habitual sleep onset and offset occurs two to three hours prior to the required sleep times. Patients with ASWD have sleep maintenance insomnia and sleepiness during the evening hours. When they are allowed to maintain an advanced schedule, sleep quality and quantity are improved [44].

Shift work sleep disorder is often experienced in the healthcare profession and often characterized by sleepiness when the individual wants to be alert, impairment in sleep quality and duration difficulty with focus/concentration, irritability and depression [45,46]. Increased cardiovascular risk and risk for malignancy, in particular breast cancer, have also been observed [47,48].

### 3.3. Treatment of Circadian Rhythm Disorders

Recent clinical practice guidelines for circadian rhythm sleep wake disorders found insufficient evidence to support efficacy of post-awakening light monotherapy as a treatment for DSPD [49]. The guidelines suggested that clinicians treat DSWD with strategically timed melatonin [49]. A study using melatonin 5 mg for 28 days revealed positive results when the dose was timed from 19:00–21:00 [50].

Recent clinical practice guidelines for treatment for circadian rhythm sleep wake disorders suggests that clinicians treat ASPD with evening light therapy and reported little to no evidence to support the use of melatonin or agonists as a treatment for ASWPD [49]. In shift work sleep disorder, consistency of work schedule (e.g., non-rotating shifts), consistency of sleep-wake times and use of sunglasses or black-out shades during the daytime when sleep is needed may be helpful strategies.

## 4. Obstructive Sleep Apnea

Obstructive sleep apnea (OSA) is characterized by recurrent episodes of partial (hypopnea) or complete (apnea) collapse of the upper airway during sleep. This results in intermittent hypoxemia, micro-arousals, sleep fragmentation, daytime sleepiness and impairment in quality of life. The prevalence of OSA in United States has increased and is estimated to be 24%–26% in men and 17%–28% in women of 30–70 years old age group [51]. The proportion of affected individuals with OSA increases with advancing age and increased body mass index. The most common presenting symptoms of OSA are daytime sleepiness, fatigue, snoring, witnessed apneas and difficulty maintaining sleep. The apnea hypopnea index (AHI) is the standard measure used to define the severity of OSA, which is calculated as the number of apneas and hypopneas that occur per hour of sleep. Even though OSA is a common condition associated with adverse consequences, it remains underdiagnosed with approximately 80% cases remains unidentified [52].

Screening for OSA can be performed using a simple screening questionnaire tool such as the STOP-BANG questionnaire [53]. A score of ≥3 of the following affirmatively indicates high pre-test probability for OSA: (1) Snoring: Have you been told that you snore loudly? (2) Tired: Are you often tired during the day? (3) Observed apnea: Do you know if you stop breathing or anyone witnessed you stop breathing while sleeping? (4) Pressure: Do you have or are you being treated for high blood pressure? (5) Body mass index: Is your body mass index >35 Kg/m^2^; (6) Age: >50 years old; (7) Neck circumference >40 cm? (8) Gender: Male.

OSA is diagnosed with in-lab polysomnography or home sleep apnea testing in those with moderate to high pre-test probability of sleep apnea and do not have significant cardiopulmonary or neurologic co-morbidities. OSA is strongly associated with variety of adverse cardiovascular outcomes including hypertension, resistant hypertension, atrial fibrillation, stroke, congestive heart failure, coronary artery disease [54] and impaired glucose intolerance, as well as non-cardiovascular outcomes including depression, motor vehicle or occupational accidents [55].

### Treatment of Obstructive Sleep Apnea

First-line treatment for OSA is positive airway pressure therapy, which operates to splint the airway to maintain patency. Meta-analyses of multiple randomized controlled trials shows significant benefit from blood pressure reduction with PAP therapy of approximately 2–3 mm Hg, albeit modest, but to a sufficient degree to result in beneficial cardiovascular outcomes. Alternative therapies for treatment for OSA include mandibular advancement devices or oral appliance therapy for those with mild to moderate OSA [56] and various surgical procedures depending on the level of collapse of upper airway such as uvulopharyngoplasty [57] and maxillomandibular advancement [58]. Novel treatment therapies such as hypoglossal nerve stimulation are also becoming increasingly considered [59].

## 5. Sleep Disorders and Dermatologic Conditions

### 5.1. Eczema and Sleep Disturbances

A recent observational study of a large population-based sample involving more than 5000 adults from 2005–2008 showed that adults with atopic dermatitis have higher odds of sleep disturbances, including difficulty sleeping and short sleep duration [60]. Children with eczema can experience intense pruritus impacting their sleep quality and quantity [61]. This has been demonstrated by objective evidence of decreased sleep efficiency measured by polysomnography and actigraphy [62]. Some data have shown that the duration of scratching episodes remains consistent in all the stages of sleep, but is more frequent in stages 1 and 2 [63] than stage 3 of sleep [64]. A number of studies have shown that actigraphy could be a viable mode of assessment of sleep in children with eczema. Actigraphic measures have been shown to be correlated with itching and quality of life in patients with eczema [65].

Various mechanisms have been postulated linking sleep disturbances and eczema, including increases in markers of systemic inflammatory cytokines and T-cell activation [66]. Interleukin-1 induced inflammation is increased in the nighttime which may play a role in enhancing nocturnal itching [67]. Furthermore, data has shown that nocturnal itching is correlated with peaks in night-time 1L-2 [68]. One of the first studies measuring actigraphic measures correlated the objective clinical scores and plasma chemokine levels in children with atopic dermatitis [69]. Table 1 demonstrates studies associating sleep disorders and eczema.

### 5.2. Psoriasis and Sleep Disorders

Psoriasis can cause insomnia symptoms such as difficulty falling asleep and maintaining sleep due to symptoms intrinsic to psoriasis [70]. On the other hand, acute sleep deprivation can intensify psoriasis inflammation in animal studies [71]. One of the potential mechanisms for this link involves dysregulation of thermoregulation and resultant compromise in the ability to disperse heat which can cause sleep interference at sleep onset [72]. Nocturnal exacerbation of pruritus in psoriasis could be explained by circadian dysfunction, *i.e.*, lower cortisol levels and decreased epidermal barrier function [73]. A recent study suggests that presence of concomitant sleep disorders in patients with psoriasis significantly increases the risk of ischemic heart disease and stroke compared to those without sleep disorders [74]. Albeit, the exact mechanisms are not known for this association, it is postulated that sleep disorders could change endocrine and metabolic profiles and sympathetic nervous activity [75] and thereby lead to increased cardiovascular mortality. A recent systematic review identified a substantially higher prevalence of OSA of 36%–82% in those with psoriasis compared to the general population and in some studies was noted to be independent of body mass index. Furthermore the authors also detected a high prevalence of insomnia which is likely facilitated by pruritus and discomfort [76].

### 5.3. Sleep and Skin Aging

A Swedish study showed that sleep deprivation affects facial cues in a negative way [77]. The sleep deprived individuals were noted to have hanging eyelids, swollen eyes, darker circles and more droopy corners of the mouth [78]. Poor sleep quality as ascertained by Pittsburgh sleep quality index >5 and sleep duration of ≤5 h was shown to be associated with increased signs of intrinsic aging and decreased skin barrier function when measured by transepidermal water loss [79]. A study assessed the effect of facial appearance after treatment of OSA with positive airway pressure therapy found that patients adherent with therapy were more attractive, and photogrammetry documented post-treatment reductions in forehead surface volume and decreased infraorbital and cheek redness [80]. On the other hand, in an animal model including elderly hairless mice, neither sleep deprivation nor sleep restriction increased any DNA damage by single cell gel assay [81]. Table 2 demonstrates studies with skin aging, facial appearance and sleep.

### 5.4. Obstructive Sleep Apnea and Skin Cancer

In a multicenter observational study involving 56 patients with cutaneous malignant melanoma, the apnea hypopnea index and oxygen desaturation index were significantly and independently associated with increased melanoma growth. In addition, these indices correlated to other aggressive markers of cutaneous malignant melanoma [82]. This study sheds light on mechanistic underpinnings of these relationships related to, for example, intermittent hypoxemia which, by means of intermediary pathways such as upregulation of hypoxia inducible factor and vascular endothelial growth factor, could enhance neovascularization of the tumor.

## 6. Conclusions

There are a broad range of sleep disorders with far-reaching implications and health effects. The high population at risk for these sleep disorders, along with the detrimental effects on quality of life and adverse health consequences, underscore the importance of expeditious diagnosis and treatment, particularly because many sleep disorders remain substantially under-recognized. Though much data has been amassed describing the ill health effects of untreated sleep disorders, it is evident that we are just scratching the surface of the interrelationships, from a mechanistic and clinical standpoint, between sleep disorders and a host of other medical disorders, including dermatological conditions.

## Figures and Tables

**Table 1:** Association of eczema and sleep disorders.

Study	Year	Ethnicity	Number of Subjects	Results
Hon	2005	Chinese	20 children	Sleep efficiency was lower in patients in severe atopic dermatitis than control group (72% *vs.* 88%)
Bender	2003	USA	14 patients with AD and 14 controls	AD group slept poorly with self-report and actigraphic measures
Bender	2008	USA	20 adults	Sleep measured by actigraphy and polysomnography were strongly associated with each other. Decreased sleep efficiency was associated with increasing disease severity, scratching, and IL-6
Hon	2006	Chinese	24 children	Wrist activities measured by DigiTrac monitor. Correlated wrist activities with disease AD severity, extent, intensity associated chemokine markers
Yu	2016	USA	5563 adults NHANES survey from 2005–2008	In multivariate regression models adults with AD had higher odds of sleep disturbances, including shorter sleep duration (OR (95% CI), 1.61 (1.16–2.25)), trouble falling asleep (OR (95% CI), 1.57 (1.10–2.24)), and early morning awakenings (OR (95% CI), 1.86 (1.24–22.78))

AD = Atopic Dermatitis, NHANES = National Health and Nutrition Examination Survey, OR = Odd’s ratio, USA = United States of America.

**Table 2:** Studies with skin aging, facial appearance and sleep.

Study	Year	Ethnicity	Number of Subjects	Sleep Disorders	Skin Features	Results
Oyetakin-White	2015	Caucasian	60 women	Sleep quality PSQI >5, sleep duration <5 h	Skin aging measured by SCINEXA^TM^ and skin barrier measured by transepidemal water loss	Lower intrinsic aging scores in good sleepers, and 30% greater barrier function compared to poor sleepers
Sundelin	2013	Swedish	20 women	Normal sleeper and 31 h of sleep deprivation followed by 5 h of sleep	40 observers rated facial photographs for fatigue, facial cues and sadness	Sleep deprived had more swollen eyes, darker circles, more wrinkles, fine lines and more droopy corners of the eyes
Chervin	2013	USA	14 men and 6 women	OSA	22 raters noted post treatment patients	Post treatment patients appeared more alert, more youthful and more attractive

OSA-Obstructive Sleep Apnea, PSQI-Pittsburgh Sleep Quality Index.
